# The global scope and components of family-centred care for preterm infants: An umbrella review

**DOI:** 10.1371/journal.pgph.0004900

**Published:** 2025-07-03

**Authors:** Jacklyn Adella, Francesca Giulia Maraschin, Shobhana Nagraj

**Affiliations:** 1 Nuffield Department of Medicine, Health Systems Collaborative, Centre for Global Health Research, University of Oxford, Oxford, United Kingdom; 2 Department of Public Health and Primary Care, Primary Care Unit, University of Cambridge, Cambridge, United Kingdom; 3 East London NHS Foundation Trust, London, United Kingdom; 4 Nuffield Department of Women’s and Reproductive Health, University of Oxford, Oxford, United Kingdom; Simon Fraser University, CANADA

## Abstract

Preterm birth is the leading cause of under-five mortality. Family-centred care (FCC) interventions may improve outcomes related to prematurity and may be used to address this issue to achieve the Sustainable Development Goals. We aimed to consolidate the scope of evidence and components of FCC interventions for preterm infants globally and see its relevance for low-resource settings. We conducted an umbrella review informed by the Joanna Briggs Institute (JBI) guidelines. Systematic literature reviews evaluating FCC in the preterm or high-risk infant population and their families were identified from six databases. Keywords included “family-centred care”, “premature infants”, “neonatal intensive care unit”, and their relevant synonyms. Quality appraisal was conducted using the JBI Critical Appraisal Checklist for Systematic Reviews and Research Syntheses and data extraction performed to an agreed table. Thematic analysis was carried out to categorise the components of FCC interventions. Forty-four reviews were included in the umbrella review. Outcomes were observed on the parents in 40 studies, the infant in 19, the health care provider in 13, and the health system in 7. Most studies focused on inpatient settings (79.6%) and were conducted primarily in high-income countries (92.3%). The components identified were general FCC, health system design, parent support, partnership in care, and information and communication. Overall, FCC interventions have a positive impact on parental, infant, and health system outcomes, with consistent reporting of FCC impact on parental well-being and satisfaction, infant length of stay, feeding and growth, and hospital readmission rates. FCC interventions have the potential to improve preterm infant health system outcomes. To maximise impact, FCC interventions need to be further explored in low-resource and post-discharge settings, where the burden of premature infant morbidity and mortality is highest. Evidence in both these settings is scarce. Future research efforts should aim to close these evidence gaps.

## Introduction

Preterm birth, defined as birth before 37 weeks of gestation, is the leading cause of under-five mortality globally, causing 17.7% of the group’s total deaths worldwide [1]. The incidence of preterm birth is estimated to affect 11% of deliveries and is increasing globally [2]. with 90% of all preterm births occurring in low- and middle-income countries (LMICs) [3]. A similar trend applies to mortality due to prematurity [4]. Addressing the burden of preterm mortality in low-resource settings is key to meeting Sustainable Development Goal (SDG) 3.2 to reduce under-5 mortality before 2030 [5].

Infants born prematurely are more likely to experience co-morbidities compared to term-born infants; some will require complex in-hospital interventions and/or special care beyond their hospital stay, and may have long-term consequences [6]. As the infant is closely connected to the family, an approach to infant care involving the family may improve infant outcomes. Furthermore, interventions that improve the physical and psychological well-being of the family may also have indirect benefits for the child [7,8]. This is especially important in low-resource settings, where access to health facilities may be limited, and thus the role of the family in infant care becomes more pronounced.

Family-centred care (FCC) is defined by the Institute of Family-Centred Care (IFCC) as “*an approach to the planning, delivery, and evaluation of health care that is grounded in mutually beneficial partnerships among health care providers, patients, and families*” [9]. This model of care emphasises collaboration between health professionals and the family in caring for the patient, and may be useful in resource-limited settings, where families have additional caregiving responsibilities resulting from workforce shortages. The Neurodevelopmental Clinical Research Unit of McMaster University bases FCC on three assumptions: 1) Parents know their children best and want the best for their children, 2) Families are different and unique, and 3) Optimal child functioning occurs within a supportive family and community context: the child is affected by the stress and coping of other family members [10]. Nine elements of FCC were described by the Association for the Care of Children’s Health, and further expanded by Trivette, et al. into 13 sub-elements, on which an intervention can be rated to determine its degree of family-centredness [11].

FCC may improve both infant and parental outcomes [12]. However, there is a broad range of interventions that embody the concept of FCC [13]. We therefore aimed to consolidate the findings of systematic reviews on FCC for preterm infants in an umbrella review, to map the scope and components of FCC interventions and how these might be translated to low-resource settings globally. Specifically, our review seeks to consolidate the existing evidence for FCC interventions and to identify knowledge gaps to inform future research.

## Methodology

### Search strategy

We conducted database searches between September and October 2022 and repeated this exercise in April 2024 using the same strategy. A definitive search strategy was developed in consultation with a librarian and database specialist. Preliminary searches were conducted in PubMed using the keywords “family-centred care” and “premature infants” to identify other keywords related to the terms in existing literature. Medical Subject Headings (MeSH terms) used in the final search strategy related to premature infants (and its synonyms), neonatal intensive care unit, family-centred care, family integrated care, patient-centred care, family nursing, nursing model, and paediatric nursing. Database-specific search strategies were constructed for each database searched: PubMed, Embase, PsycInfo, Web of Science, CINAHL, and Cochrane database of systematic reviews (see Supplementary material). Following screening for eligible papers, we conducted citation searches.

### Eligibility criteria

A summary of this umbrella review’s eligibility criteria can be found in the Supplementary material.

### Study type

Studies were eligible for inclusion if they conducted and presented the results of a systematic search of the literature, including systematic reviews, meta-analyses, meta-ethnographies, meta-syntheses, mixed-method reviews, and integrative reviews. Articles categorised as guidelines or recommendations were eligible as long as the literature search was conducted systematically and the results of the evidence were presented in the article.

### Population

Studies investigating preterm infants, families and/or caregivers of the preterm infants, and healthcare professionals involved in the care of preterm infants were included. We further included studies involving Neonatal Intensive Care Unit infants or “critically sick babies” and their families as the population of interest. We included these studies with the assumption that a significant proportion of this population would have been premature infants, and that FCC applied to a similar population may yield similar outcomes [14,15].

### Concept

We included studies that investigated an intervention or phenomenon that we categorised as FCC for preterm infants and their families, whether on its own or alongside other interventions. We used a broad approach to determine which intervention/phenomenon could be classified as family-centred. Interventions that fulfilled any of the elements of FCC from Trivette, 1993 [11] were included. We also included studies investigating the needs of parents and/or healthcare professionals in the context of caring for premature infants, barriers and facilitators of FCC, and perspectives regarding FCC interventions, as these studies could inform future implementation of FCC. We excluded studies exclusively reviewing kangaroo care or an equivalent intervention involving skin-to-skin contact, as we felt that this is a well-researched area with clear benefits [16,17], and the number of studies appraising this particular intervention would overshadow other health system-wide FCC interventions.

### Context

We included studies from all contexts (in health facilities and at home or community, and all income settings).

### Exclusion criteria

Primary studies, reviews that did not employ a systematic search method, and studies published not in English were excluded.

### Study selection

All identified citations were imported into EndNote 20 (Clarivate, 2013). Duplicates were removed. De-duplicated search results were then uploaded to Rayyan© (rayyan.ai), a web-based tool to conduct systematic reviews [18]. Titles and abstracts were screened using the eligibility criteria, and full texts of eligible studies were screened using Rayyan by two reviewers (JA, FM). Disagreements between reviewers were discussed with a third reviewer (SN). Database searches were supplemented by citation searching. This process was summarized according to the PRISMA 2020 guideline [19].

### Data extraction

We created a data extraction table based on the Joanna Briggs Institute (JBI) guide for umbrella reviews [20] which was modified and agreed between the authors. The data extracted included authors, year published, title, type of review, intervention/objective, number and type of primary studies, country, participant population, setting of interest, components of FCC intervention, and main outcomes. Data extraction was conducted using Microsoft Excel v. 2301 by JA and verified by FM.

### Quality appraisal

Quality of included studies was conducted independently by two reviewers (JA & FM) using the JBI Critical Appraisal Checklist for Systematic Reviews and Research Syntheses [20]. The checklist includes 11 items, for which each was marked as “Yes”, “No, “Unclear, or “Not applicable.” An overall quality rating (high, moderate, low, or unclear) was assigned based on the pattern of responses and comparison with other included reviews. Discrepancies were resolved through discussion. The full set of quality appraisal results is available in the Supplementary material.

### Data syntheses

We conducted a thematic content analysis of included studies, inductively deriving themes based on the objectives of the review, with the aim of identifying the scope of FCC interventions. Derived themes were discussed between the review team to reach a consensus. Due to the heterogeneity of the reviews, the findings were narratively synthesised.

## Results

### Results of the search

**Fig 1:**
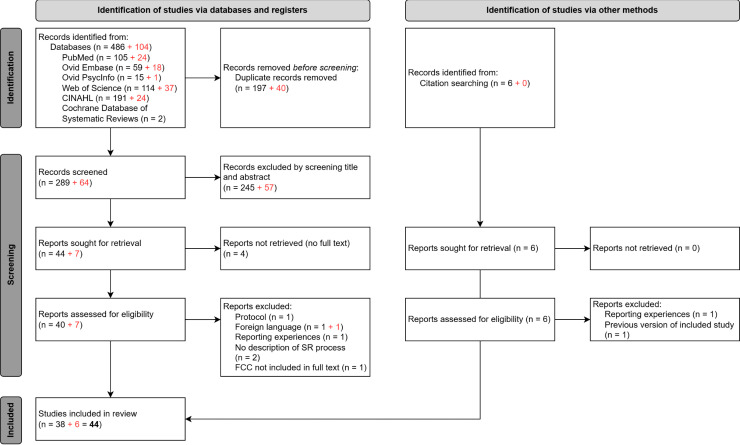
PRISMA 2020 flow diagram of search results, excluded, and included records. The result of the search conducted in April 2024 is indicated in red.

The initial search yielded 486 records from six databases. After de-duplication, 289 titles and abstracts were screened, and a total of 40 full-text papers were assessed for eligibility. Out of these, 6 papers were excluded. Citation searching yielded a further 4 papers. In 2024, the search yielded a further 104 new records, 40 of which were duplicates. Sixty-four titles and abstracts were screened, and 7 full-text papers were assessed for eligibility. One paper was excluded. Citation searching yielded no additional papers. A total of 44 papers were therefore included in the umbrella review. The PRISMA diagram of this process is illustrated in Fig 1 [19].

### Characteristics of included reviews

There was considerable heterogeneity among the included studies. Studies varied in terms of review design, population of interest, range of interventions, and outcomes. We classified 3 (6.8%) as integrative reviews and 41 (93.2%) as systematic reviews. Out of the systematic reviews, 19 (46.3%) were of quantitative studies only, 8 (19.5%) of qualitative studies, and 14 (34.2%) included a mix of quantitative and qualitative studies. Nine papers conducted a meta-analysis.

We defined the population of interest as the population in which the outcomes were observed. Most reviews had more than one population of interest. The infant was the population of interest in 19 reviews, caregiver/parent in 40, health care provider (HCP) in 13, and the health system in 7 studies.

The setting of interest also varied. Thirty-five reviews (79.6%) focused on inpatient settings (most commonly NICUs), 2 reviews (4.6%) on other healthcare settings, 3 (6.9%) on home or community, and 4 reviews (9.1%) did not specify their setting of interest or were interested in all settings. The latter reviews focused on interventions or phenomena that were not bound to a specific setting, although most primary studies were undertaken in a hospital.

Most reviews (n = 39, 88.6%), reported details of the countries in which the primary studies were conducted. Out of these, 36 reviews (92.3%) included primary studies only or predominantly from high-income countries (HICs), while 3 reviews (7.69%) had primary studies only or predominantly from LMICs. Ten reviews had primary studies exclusively from HICs. Four reviews included primary studies conducted in low-income countries (LICs) in their analyses.

### Quality evaluation of the reviews

The majority of included reviews had strong methodological quality (n = 31, 70.5%). Seven (15.9%) were marked as moderate quality, 5 (11.4%) as weak quality, and 1 (2.3%) as unclear quality. Most reviews limited their inclusion criteria to several publication languages (most only included studies in English and several other languages). Many did not include grey literature. Most did not assess the likelihood of publication bias.

### Findings of the umbrella review

**Fig 2:**
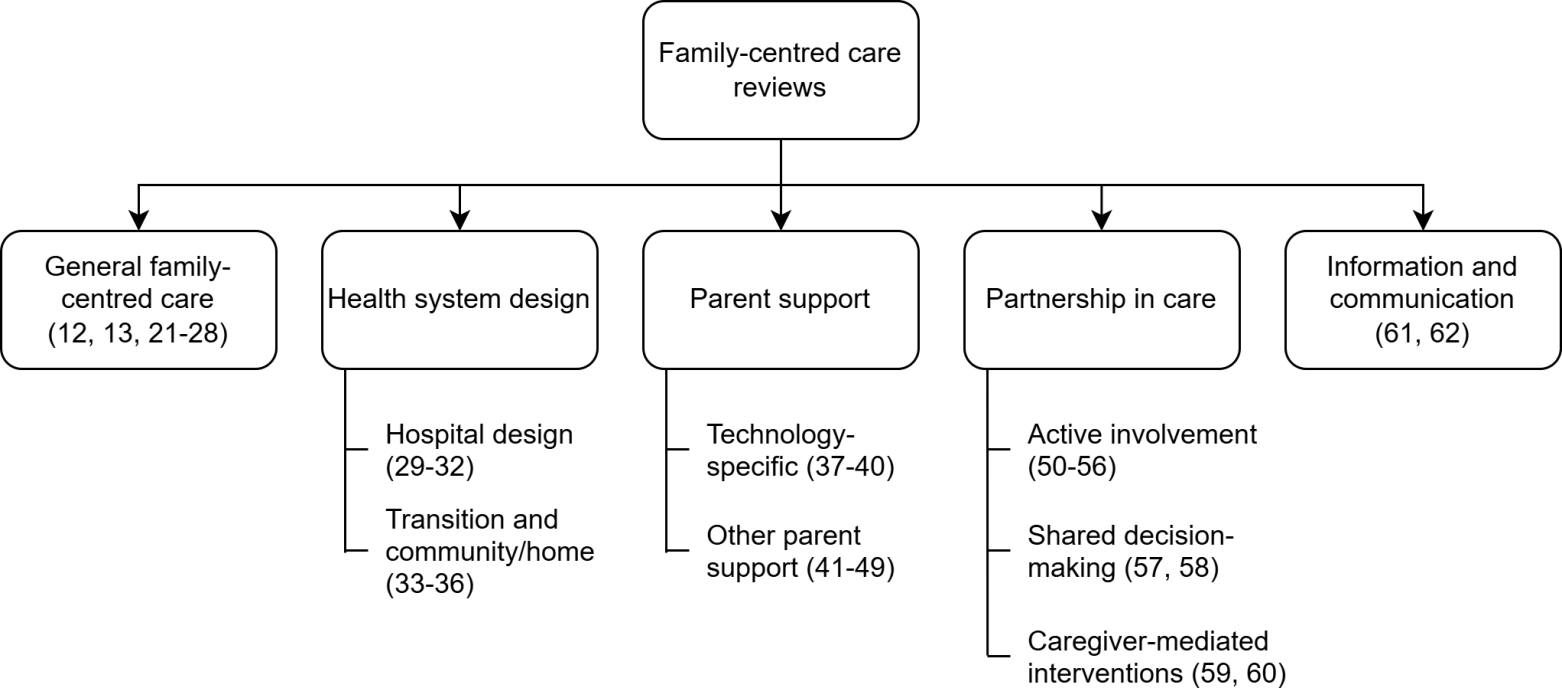
Thematic categorization of FCC interventions based on included reviews. Reference numbers (in brackets) correspond to the numbered bibliography.

The results of our thematic analysis are presented in Fig 2 and a summary of outcomes in Table 1. The complete data extraction table can be seen in the Supplementary material.

**Table 1:** Summary of family-centred care interventions and outcomes.

Theme/subtheme	Infant outcome	Parental outcome				Health system outcome	Note
		Well-being	Satisfaction	Skills and knowledge	Involvement and collaboration with HCPs		
General FCC	^LOS generally shortened, better feeding and growth, outcomes of some morbidities improved, readmission rate lowered. Difference in neurobehavioural performance not significant.	^Lower anxiety, depression, and stress levels. Family access and participation restrictions led to acute distress in parents.	^Increased	^FCC empowers parents, increases nursing skills, knowledge, and self-efficacy	^Family access and participation restrictions lead to parent-parent and parent-infant separation		
Health system design: hospital design	^CBPU/SFRs increases sleep, reduces risks of infection, increases physiological stability. Transitional wards shortens LOS, reduces risks of cross-infection.	^Parental stress lower. No differences found for anxiety.	^Increased	*No differences for self-efficacy	*No differences found for parent-infant bonding. ‘Mother as main carer’ model enabled.	^Cost of CBPU lower compared to standard design. Infants less likely to receive inadequate care	Physicians more supportive of SFR design compared to nurses. Parental involvement critical in single family room design
Health system design: transition and community/home	^Shorter LOS. Benefits for children development unclear. Lower readmission rates.	^Maternal well-being improved.	^Increased	^Parenting improved			
Parent support: technology specific	*LOS and postmenstrual age at discharge lower, but not significant.	*Reduced worry/anxiety and stress. Some interventions may cause some parents increased anxiety and hypervigilance.	*No significant change in parental satisfaction	^Parental responsiveness and empowerment increased	^Promotes parent-infant bonding, fosters collaboration between HCPs and patients’ families at home.	*No significant financial impact	Intervention infrastructure has to function properly and be well-planned
Parent support: other	^Mental health interventions had positive impact on infant mental development. Some aspects of infant feeding were improved, but no significant impact on time to full oral feeding or to complete feeds. No efficacy on LOS.	^Different parental support strategies can reduce long-term parental distress. Mental health interventions have positive impact on maternal anxiety and coping, but no efficacy on QoL and physical health. Psychospiritual and psychosocial interventions improve parental well-being.	^Mental health interventions has positive impact on parental satisfaction	^Mental health interventions has positive impact on parental confidence			Parents experience significant emotional distress. They have multifaceted needs, predominantly around emotional, informational, and practical support.
Partnership in care: active involvement	^Positive impacts on breastfeeding, earlier weight gain, lower rates of infection, better neurobehavioural outcomes, some measures of infant growth, and lowers risk of retinopathy of prematurity. No impact on mortality, risk of bronchopulmonary dysplasia, necrotising enterocolitis, and intraventricular hemorrhage.	*Positive impact on maternal mental health and well-being, parental stress and anxiety levels, and maternal health behaviour. May increase some parents’ stress and feeling of helplessness.	^Increased	^Parental self-efficacy higher	^Better maternal-child bonding	^More humanized care in the hospital, higher follow-up appointment attendance	Effective communication, trust, information sharing, support from the HCPs, and healthcare policies and infrastructure needed to allow parental participation in hospital care.
Partnership in care: shared decision making							Barriers and facilitators exist to shared decision making between parents and HCPs.
Partnership in care: caregiver-mediated interventions	*Mixed results, although some show positive outcomes: lower pain parameters, improved cognitive development, shorter LOS	^Improved maternal well-being (distress, depression, anxiety)	^Increased	^Increased confidence			
Information and communication		^Positive impact on coping	^Increased	^Positive impact on knowledge and parenting	^Positive impact on parental participation		Information and communication positively impact parental outcomes, and vice versa.

The categories of FCC interventions and their outcomes. All outcomes refer to the intervention group compared to the control group. FCC: family-centred care, HCP: health care provider, LOS: length of stay, CBPU: care-by-parent-unit, SFR: single family room.

^indicates the presence of at least one positive outcome with/without some outcomes that were found not statistically significant.

*indicates an overall negative, conflicting, or uncertain outcome.

### General family-centred care

The theme general FCC encompassed reviews that looked at a group of interventions that fulfilled a predetermined set of FCC criteria. Three out of the nine reviews in this theme [21–23] used the family-centredness scoring by Trivette [11] to determine the inclusion of primary studies. Segers et al. and Barnes et al. determined their inclusion criteria based on the core concepts of FCC (dignity and respect, information sharing, participation, and collaboration of families/parents) [24,25]. Davidson et al., Ding et al., and McAndrew et al. developed their own inclusion criteria for FCC interventions [12,26,27]. Van Veneendaal et al. looked at the impact of the restriction of family-centred care measures in neonatal care due to the Covid-19 pandemic [28]. Brett et al. mapped out effective interventions for communicating, supporting, and providing information to parents [13].

The primary outcome most frequently reported in these studies was length-of-stay (LOS). FCC generally shortened the hospital and/or NICU LOS, although its significance differed between reviews [12,21,24,27]. Ding et al. [12] found the mean difference of hospital LOS to be -3.73 (95% CI, -9.25 to 1.79), while Yu and Zhang [21], whose primary studies mostly overlapped with those of Ding et al., [12] found the mean difference of hospital LOS to be -4.77 (95% CI, -8.77 to -0.76) and NICU LOS to be -4.62 (95% CI, -7.40 to -1.83). Segers et al. [24] did not conduct a meta-analysis, but found that four out of seven primary studies showed statistically significant reduction of hospital/NICU LOS. Infants cared for using an FCC model reported better feeding and growth outcomes [12,21,27], a significantly reduced risk of moderate to severe bronchopulmonary dysplasia [21], and readmission rate [12]. One review assessed neurobehavioural performance, but the difference was found to be not significant [12]. Impact on other comorbidities, such as sepsis, was not significant [21].

Parental satisfaction was found to be consistently higher in FCC intervention groups [12,24,25,27]. FCC seemed to empower parents [27], increased their nursing skills [12], knowledge [12], and self-efficacy [27]. Parents in FCC intervention groups mostly demonstrated lower anxiety, depression, and stress levels, although the statistical significance varied between studies [12,25,26].

Van Veenendaal et al. [28] found that Covid-19 severely restricted family access and participation in infant caregiving. Restrictions to access as a result of the pandemic led to increased parent-infant and parent-parent separation, as well as acute distress in parents [28]. Watts et al. [23] found that parents of a hospitalised child wished to be involved in the care of their child. Furthermore, the parents themselves may also need care in the NICU setting [23]. Improving interpersonal skills, effective communication by the health care professionals, as well as creating opportunities for FCC were highlighted as effective strategies to improve parental satisfaction [23].

Brett et al. grouped effective interventions for supporting parents into the following areas: 1) individualised developmental and behavioural care programmes, 2) behavioural assessment scales, 3) breastfeeding, KC, and infant-massage programmes, 4) support forum for parents, 5) alleviation of parental stress, 6) preparing parents for seeing their infant for the first time, 7) communication and information sharing, 8) discharge planning, and 9) home-support programmes [13].

### Health system design

#### Hospital design.

Four reviews addressed hospital design interventions. Kuhn et al. [29] and van Veenendaal et al. [30] compared single-family rooms (SFRs) and open bay units in the NICU setting. Similarly, Shields et al. reviewed one study that examined the effect of a care-by-parent unit (CBPU, a unit where parents were accommodated and encouraged to participate in the care of the child) compared to standard hospital admission [31]. SFRs benefited preterm infants by providing acceptable sound simulation, increasing sleep, reducing risks of infection, and increasing physiological stability, but highlighted that the involvement of parents was critical in this design [29]. The review by Shields, et al. [31] reported that children cared for in a CBPU were less likely to receive inadequate care. However, this study did not find any significant differences in behavioural or physical outcomes [31].

SFRs/CBPU supported parental involvement in the care of the infant, as well as reduced parental stress and increased parental satisfaction [29–31]. There were no differences found for anxiety, parent-infant bonding, and self-efficacy [30]. HCPs recognised the value of SFRs, but physicians were more supportive of the design than nurses [29]. The cost of CBPU care was lower compared to standard inpatient admission [31].

Miah examined the benefits of NICU-standard ward transitional care in settings similar to the UK [32]. Transitional wards shortened LOS and enabled the ‘mother as main carer’ model of care, reducing risks of cross-infection.

#### Transition and community/home.

Four reviews examined the transitions of healthcare between the formal health system and the community. Pladys et al. [33] reviewed the hospital to home transition, Menczykowski et al. [34] reviewed early discharge programs for tube-fed preterm infants, Kearney et al. [35] reviewed effects of home visits in the US and Canada, and Benzies et al. [36] reviewed early intervention programs.

Overall, more family-centred, non-hospital-based health system designs, had positive impacts for the infant in relation to shorter LOS and lower readmission rates [33], and appeared to be safe [34], although benefits for child development were unclear [35]. The FCC interventions improved maternal well-being (including stress, anxiety, and depressive symptoms) [35,36], parental satisfaction [34], sensitivity/responsiveness [36], and self-efficacy [36]. Effects on parental attitudes and behaviors (measured by various tools, including the Home Observation for Measurement of the Environment – HOME, other scales, and reduction of injuries, emergency room visits, or reported child abuse or neglect) were mixed [35].

### Parent support

#### Technology specific.

Four reviews examined technology-specific interventions to support parents. Dol et al. [37] examined technology-based health (eHealth) interventions for education and/or communication, Gibson and Kilcullen [38] examined web-camera to send live view of infants, and Koh et al. [39] examined audio recordings of consultations with doctors. All three reviews were conducted in a NICU setting, but Koh et al. [39] did not find any primary study fulfilling their inclusion criteria. Nordtug et al. [40] examined the use of videoconferencing (VC) by HCPs for patients and their families in home-care situations and post-hospital discharge follow-up at home.

In general, the use of technology-based interventions seemed to be well accepted by parents and feasible, with certain conditions: network access and the technology have to function properly and the intervention has to be well-planned [37,40]. There were no hard outcomes reported from the papers in the technology-specific interventions subtheme, although intervention groups showed a moderate, but non-significant, improvement in LOS and postmenstrual age at discharge [37]. Technology-based interventions may benefit parents as they promoted parent-infant bonding, increased parental responsiveness, reduced worry/anxiety and stress, and increased parents’ feelings of empowerment [37,38,40]. Technology-mediated communication could foster collaboration between HCPs and patients and/or patients’ families at home [40]. Dol et al. [37] found no significant financial impact and parental satisfaction from eHealth interventions. Additionally, some technology-based interventions caused parents to experience increased anxiety and hypervigilance of their infants [38].

#### Other parent support.

There were nine reviews in this subtheme. The needs of parents of premature or critically sick infants were examined by Cleveland [41], Mousavi et al. [42], and Hurt et al. [43] Strategies (and their effects) to support parents were assessed by Cleveland [41], Maleki et al., [44] Sabnis et al., [45] and Holm et al. [46] Kasparian et al., [47] Brelsford et al. [48], and Chan and Shorey [49] specifically examined outcomes of mental health interventions, psychospiritual interventions, and psychosocial interventions, respectively.

Parents of premature or critically sick infants have multifaceted needs, predominantly around emotional, informational, and practical support [41–43]. They experience significant emotional distress, including anxiety, stress, and feelings of helplessness, often exacerbated by societal stigma and the unexpected nature of their infant’s condition [41,43]. Addressing the parents’ needs through strategies including providing emotional support, creating a welcoming healthcare unit environment, and parent empowerment, including facilitating skin-to-skin contact between the infant and the parent, could reduce long-term parental distress [41,44–46].

A review of mental health interventions showed a positive impact on maternal anxiety and coping, mother-infant attachment, parental confidence and satisfaction, and infant mental development; while the effects on depression, infant feeding, quality of life, physical health, and LOS were mixed or insignificant [47]. Psychospiritual interventions improved parental well-being by way of less stress and higher quality of life scores [48]. Psychosocial interventions reduced symptoms of stress, depression, and anxiety [49].

### Partnership in care

#### Active involvement.

Seven reviews examined active involvement of parents in the care of the infant. Brødsgaard et al., [50] Chan et al., [51] and Gómez-Cantarino et al. [52] examined the views of parents and HCPs regarding parent involvement in infant care in the NICU. Gómez-Cantarino et al., [52] Klawetter et al., [53], Shields et al., [54] North et al. [55], and Kutahyalioglu and Scafide [56] examined outcomes of parental active involvement.

The reviews examining the perspectives of parents and HCPs regarding parental involvement yielded contradicting results. On one hand, parental participation in the care of their baby allowed for improving their knowledge, enabled task-sharing between parents and HCPs, and helped to relieve parental stress. However, parents may feel stress and helplessness when seeing their infants undergo painful procedures [50–52]. Additionally, to enable parental participation in the NICU/for hospitalised preterm infants, requirements such as effective communication, mutual trust, information sharing, support from the HCPs, and the necessary healthcare policies and infrastructure are required [50,51].

Active involvement of parents in the NICU was found to have generally positive outcomes for the infant, parents, and health system. Active involvement of parents in the NICU was associated with positive impacts on breastfeeding [52,53,55], earlier weight gain [52,55], better neurobehavioural outcomes [52,53,55], shortening of hospital LOS (although not NICU LOS) [53,55], and decreased risk of retinopathy of prematurity [55]. Being held by parents during transfer led to more stable vital signs in the infant [53], while active involvement in general promoted better maternal-child bonding, although no meta-analysis was conducted [53,56]. Active involvement of parents did not have a significant impact on infant mortality [52,55], infection during hospitalisation [52,55], morbidities including necrotising enterocolitis, bronchopulmonary dysplasia and other respiratory outcomes, and intraventricular hemorrhage [55].

Parental outcomes reported were around maternal mental health and well-being, for which studies reported a positive impact [52–55]. Parental stress and anxiety levels were found to be lower, although some primary studies reported no significant difference [52,53,55]. The following were also found in the intervention group, but no meta-analysis was performed: better maternal health behaviour outcome [53], higher parental self-efficacy and satisfaction [52,54], and more humanized care in the hospital, as well as higher follow-up appointment attendance [52,53].

#### Shared decision making.

Both papers that reviewed shared decision-making (SDM) looked at barriers and facilitators in implementing SDM. Parish et al. looked at parental perceptions, while Légare et al. looked at HCP’s perceptions [57,58]. From the parents’ perspective, key barriers to SDM were emotional overwhelm, inadequate information and communication, lack of continuity in care or caregivers, and power imbalances between parents and HCPs; while key facilitators were good communication, continuity in care and caregivers, caring HCPs, and tailored decision-making approach [57]. From the HCP perspective, key barriers to SDM were a lack of time and a lack of applicability due to patient characteristics or clinical situation; while key facilitators were motivation of HCPs and the perception that SDM had positive impacts [58]. No reviews that looked for outcomes of SDM were found.

#### Caregiver-mediated interventions.

The reviews in this subtheme included interventions that were mediated by caregivers. One review appraised parental involvement in pain management [59] and one reviewed general caregiver-mediated interventions [60].

Fiest et al. found that for studies on the infant population, caregiver-mediated interventions had almost equal numbers of studies that showed significant positive outcomes compared to insignificant or mixed results outcomes on the infant and parents [60]. Significant positive outcomes reported were maternal well-being, confidence, and satisfaction, shorter infant NICU and hospital LOS, as well as infant cognitive development, although other studies showed no significant differences in some of the outcomes mentioned [60]. Eissler et al. found that caregiver-mediated interventions, including kangaroo/skin-to-skin care, facilitated tucking, and breastfeeding, lowered pain parameters during painful interventions on the infant [59].

### Information and communication

Two reviews were classified under the theme information and communication. Wreesmann et al. analysed the functions of adequate communication and its essential factors [61]. Labrie et al. synthesised the effect of parent-provider communication in the NICU on parent-related outcomes [62].

According to Wreesmann et al., the functions of communication in the NICU were to build and maintain relationships, to exchange information for SDM, and to enable parent self-management. Adequate communication in the NICU entailed HCP’s attention to the topic, communication aims, location, route, and design of the interaction [61]. Labrie et al. found that positive communication experiences affected several parental domains positively, namely coping, knowledge, participation (in communication and in care), parenting (empowerment and parent-infant attachment), and satisfaction, and vice versa [62].

## Discussion

In this umbrella review, we outline the scope and components of FCC interventions for preterm infants, which we thematically grouped into general FCC, health system design, parent support, partnership in care, and information and communication interventions. Papers included in our umbrella review synthesized data in different ways, including mapping out intervention components and their functions, exploring the acceptability, barriers, and facilitators to FCC-type interventions, and exploring the needs and experiences of caregivers, as well as investigating the intervention impact on clinical and health-system outcomes.

Overall, the studies included in the umbrella review reported positive impacts on parental well-being, satisfaction, parenting skills, and parent-infant bonding [12,13,23–25,27–31,33–38,40,44–49,52–56,60,62]. There were consistent benefits on infant outcomes, such as LOS, feeding and growth, and hospital readmission rates [12,21,24,27,29,32–35,37,53,55]. Impacts on cause-specific morbidity and mortality were less consistent or less reported. Few studies reported the impact of FCC intervention on health systems, but they showed that FCC may have benefits for the health system [31,40,54,60]. A well-designed and implemented FCC programme is needed to bring about positive outcomes, while a poor one may cause harm [62].

Our review showed that FCC can contribute positively to reducing the length of stay [12,21,24,27,32,33,37,53,55]. While the significance of this reduction varied between reviews, mean differences by Ding et al., [12] Yu and Zhang [21], and Segers et al., [24] found an overall significant reduction in LOS where FCC interventions were introduced. These findings are echoed in the wider literature. In a study by Ortenstrand et al., measuring the effect of parental involvement on the LOS for neonates in NICU, it was concluded that hospital stay was reduced by 5.3 days (p < 0.05) [63]. The consistent evidence underscores the pivotal role of FCC in optimizing patient outcomes by shortening hospital stays.

Another important finding in our review was that parental satisfaction and self-efficacy were reported to be consistently higher in groups where FCC interventions were introduced [12,24,25,27,30,34,36,47,54]. Targeted health system designs, such as SFRs and CPBUs, enhanced parental satisfaction while costing less than standard inpatient admission [29–31]. Where parents were involved in active care, stress levels were lower and parents reported feeling empowered [52–55]. This is important given that parental efficacy is crucial for the well-being of both parents and children [64]. Furthermore, increasing parental participation in care, employing caregiver-mediated interventions such as skin-to-skin contact, information dissemination to parents, and improving parent-provider communication have all been previously deployed and researched to some extent in this setting, and have shown positive outcomes on neonatal well-being [65–68].

As previously noted, LMICs account for a large majority of the global preterm births and neonatal deaths due to prematurity [3,4]. Improving care for preterm infants in these settings is critical to achieving global child survival targets. FCC may offer a promising approach to address systemic resource constraints by leveraging an underutilized human resource (the family) to help fill gaps in newborn care, thereby improving neonatal outcomes. Furthermore, FCC aligns with the principles of equity and respectful care by treating parents as partners in caregiving. As shown in this review, a range of parental outcomes, including well-being, satisfaction, and efficacy, tended to improve with FCC interventions, which may enhance trust and satisfaction in healthcare services even in resource-constrained settings.

The range of FCC interventions described in our study can potentially be deployed in low-resource settings. However, more studies conducted in the relevant socioeconomic context, particularly those assessing cost-effectiveness, are needed to ensure that they are appropriately designed and implemented to achieve their intended benefits without causing harm. This is especially important given that the complex nature and interactions involved in FCC interventions cannot always be clearly defined. Literature has emphasised the importance of designing and articulating the complexity of interventions and considering their mechanisms of impact through developing program theory and measuring their anticipated effects [69,70].

### Limitations

We found significant heterogeneity across studies in terms of the types of interventions, study populations, and reported outcomes. There were few reviews that appraised FCC as an “intervention package”. Indeed, the scant number of reviews following strict FCC inclusion criteria [21,22,31,54] found very few to no primary studies meeting these criteria. Studies varied in their definition of FCC and there was little consensus identified on how to measure the “dose” of FCC-type interventions. Furthermore, few studies reported the impact of FCC interventions on clinical outcomes and rather focused on processes of care and care quality. These factors made it difficult to draw conclusions on the effectiveness of FCC interventions on specific clinical outcomes that might impact neonatal or infant mortality.

The majority of the existing literature on FCC interventions for preterm infants was in the context of the hospital NICU environment and focused on immediate/acute outcomes. Few studies addressed care for preterm infants outside the context of NICU, such as in transitional wards, and even fewer in the community setting. Most neonatal deaths will happen at home after discharge from a healthcare environment, and the consequences of prematurity may be long-term, such as disrupted physiological and neurodevelopmental growth [6,71,72]. To ease neonatal morbidity and mortality, FCC is one potential way to empower parents/caretakers to better care for the infants at home, for a considerably longer period as opposed to the acute hospital stay [35]. Our study showed that FCC can empower parents, as well as increase their skills and knowledge. FCC in the community can potentially address this issue, and future research should aim to generate evidence for this prospect. These findings are supported by the wider literature on community/home-based care for infants [73–75].

Furthermore, although the majority of preterm morbidity and mortality happen in low-resource settings [3], most of the data included in the reviews were obtained from HICs. Included reviews which did not limit their inclusion language found very few non-English primary studies on the topic [23,28,30]. Future research should be focused on generating evidence from low-resource settings to further establish the need, effectiveness, and feasibility of FCC implementation in this environment.

To our knowledge, this is the first umbrella review on FCC for preterm infants. Our review used a broad definition of FCC, allowing us to see the breadth of available evidence that can contribute to this topic. Due to limited human, time, and financial resources, we limited our search to literature published in English and we did not contact authors for full-texts. We did not assess for publication bias.

## Conclusion

Preterm birth has long-lasting impacts on the infant and their family, and may benefit from FCC interventions. This umbrella review reports findings from 44 systematic and integrative reviews to understand the scope and components of FCC interventions for preterm infants. We found that a broad range of aspects and components of FCC interventions had been researched. FCC interventions seemed to have positive impacts on infant, parental, and health system outcomes, but the evidence was often inconclusive due to the heterogeneity of the studies. There were gaps in the literature for FCC interventions carried out in the home or community, and in low-resource settings. Future research efforts should aim to 1) better define FCC interventions, 2) determine causal or strong correlative relationships using strong study designs, and 3) address the lack of evidence for community-based FCC in low-resource settings.

## Supporting information

S1 Table Eligibility criteria. (DOCX)

S2 Table Databases searched and search strategy. (DOCX)

S1 File Data extraction table and quality appraisal summary. (XLSX)

S2 File PRISMA 2020 checklist. (PDF)

S3 File PRISMA 2020 for abstracts checklist. (PDF)
