# Supplementary material for: The global scope and components of family-centred care for preterm infants: An umbrella review
Source: PLOS Glob Public Health. 2025 Jul 3;5(7):e0004900. doi: 10.1371/journal.pgph.0004900 (PMC12225843; doi:10.1371/journal.pgph.0004900)
Supplement: S1 Table — (DOCX) [file pgph.0004900.s001.docx]

# Eligibility criteria

Umbrella Review of Family-Centred Care for Preterm Infants
Adella J, Maraschin FG, Nagraj S

| Study type | Population | Concept |
| --- | --- | --- |
| INCLUSION CRITERIA | | |
| All literature reviews conducted systematically, including those embedded in recommendations or guidelines:  Systematic reviews, meta-analyses, meta-ethnographies, meta-syntheses, mixed method reviews, integrative reviews | Preterm infants  Any population involved in the care of preterm babies OR babies in the NICU | Investigates an intervention categorised as family centred care, fulfilling any of the following characteristics (Trivette, 1993):   - Family as a constant - Facilitating parent-professional collaboration - Honoring family diversity (racial, ethnic, cultural, socioeconomic diversity - Recognizing family strengths and individuality and respecting different methods of coping - Sharing complete and unbiased information with families on a continuous basis - Encouraging/facilitating family-to-family support - Responding to child and family developmental needs - Adopting policies and practices that provide families with emotional and financial support - Designing health care that is flexible, culturally competent, and responsive to family needs   OR studying something that directly informs the implementation of family-centered care (e.g. needs of parents, views on facilitators and barriers of FCC) |
| EXCLUSION CRITERIA | | |
| - Primary studies - Protocols - Theoretical studies, concept analyses - Published opinions - Studies in other languages than English - No full-texts |  | - Studies that only have FCC as a result or recommendation - Studies that appraise strictly general perspectives of target population on standard care - Kangaroo care or equivalent skin-to-skin interventions |
