# Supplementary material for: The global scope and components of family-centred care for preterm infants: An umbrella review
Source: PLOS Glob Public Health. 2025 Jul 3;5(7):e0004900. doi: 10.1371/journal.pgph.0004900 (PMC12225843; doi:10.1371/journal.pgph.0004900)
Supplement: S2 Table — (DOCX) [file pgph.0004900.s002.docx]

# Databases searched and search strategy

Umbrella Review of Family-Centred Care for Preterm Infants
Adella J, Maraschin FG, Nagraj S

| Database | Query | Hits - 14 Oct 2021 | Hits - 20 Apr 2024 (new) |
| --- | --- | --- | --- |
| PubMed | (((((("Infant, Premature" [MeSH]) OR ((Preterm OR premature) AND (Infant* OR neonate* OR baby OR babies))) OR ("neonatal prematurity"))) OR ("Intensive Care Units, Neonatal"[Mesh])) OR ("Newborn Intensive Care Unit*" OR "Neonatal Intensive Care Unit*" OR NICU OR "Neonatal ICU*" OR "Newborn ICU*")) AND ((("family-centered" OR "family-centred" OR "family centered" OR "family centred" OR "family integrated care" OR "patient-centred care" OR "patient centred care" OR "patient-centered care" OR "patient centered care" OR "family nursing" OR "family-centred nursing" OR "family centered nursing" OR "maternal child nursing" OR "pediatric nursing" OR "paediatric nursing" OR "nursing model*" OR FCC)) OR (("Family"[Mesh]) AND "Patient-Centered Care"[Mesh]))) AND (((((systematic*[tiab] AND (bibliographic*[TIAB] OR literature[tiab] OR review[tiab] OR reviewed[tiab] OR reviews[tiab])) OR (comprehensive*[TIAB] AND (bibliographic*[TIAB] OR literature[tiab])) OR "cochrane database syst rev"[Journal] OR "Evidence report/technology assessment (Summary)"[journal] OR "Evidence report/technology assessment"[journal] OR "integrative literature review"[tiab] OR "integrative research review"[tiab] OR "integrative review"[tiab] OR "research synthesis"[tiab] OR "research integration"[tiab] OR cinahl[tiab] OR embase[tiab] OR medline[tiab] OR psyclit[tiab] OR (psycinfo[tiab] NOT "psycinfo database"[tiab]) OR pubmed[tiab] OR scopus[tiab] OR "web of science"[tiab] OR "data synthesis"[tiab] OR meta-analys*[tiab] OR meta-analyz*[tiab] OR meta-analyt*[tiab] OR metaanalys*[tiab] OR metaanalyz*[tiab] OR metaanalyt*[tiab] OR "meta-analysis as topic"[MeSH:noexp] OR Meta-Analysis[ptyp] OR ((review[tiab] AND (rationale[tiab] OR evidence[tiab])) AND review[pt]))) | 105 | 129 (24) |
| Ovid Embase | Database: Embase 1974 to present  Search Strategy:  --------------------------------------------------------------------------------  1 exp Meta Analysis/ (259891)  2 ((meta adj analy$) or metaanalys$).tw. (316597)  3 (systematic adj (review$1 or overview$1)).tw. (315107)  4 or/1-3 (522240)  5 cancerlit.ab. (738)  6 cochrane.ab. (153621)  7 embase.ab. (171272)  8 (psychlit or psyclit).ab. (1004)  9 (psychinfo or psycinfo).ab. (50482)  10 (cinahl or cinhal).ab. (48233)  11 science citation index.ab. (4125)  12 bids.ab. (808)  13 or/5-12 (263948)  14 reference lists.ab. (22633)  15 bibliograph$.ab. (27240)  16 hand-search$.ab. (10024)  17 manual search$.ab. (6631)  18 relevant journals.ab. (1548)  19 or/14-18 (61400)  20 data extraction.ab. (35795)  21 selection criteria.ab. (42679)  22 20 or 21 (75928)  23 review.pt. (2959540)  24 22 and 23 (35608)  25 letter.pt. (1241775)  26 editorial.pt. (739421)  27 animal/ (1589954)  28 human/ (24032348)  29 27 not (27 and 28) (1165055)  30 or/25-26,29 (3128074)  31 4 or 13 or 19 or 24 (616937)  32 31 not 30 (601158)  33 prematurity/ (118937)  34 newborn intensive care/ or neonatal intensive care unit/ (45149)  35 (((Preterm or premature) adj4 (Infant* or neonate* or baby or babies)) or "neonatal prematurity" or "Newborn Intensive Care Unit*" or "Neonatal Intensive Care Unit*" or NICU or "Neonatal ICU*" or "Newborn ICU*").ti,ab,kw. (108536)  36 33 or 34 or 35 (177849)  37 family/ (99888)  38 patient care/ (337638)  39 37 and 38 (4616)  40 ("family-centered" or "family-centred" or "family centered" or "family centred" or "family integrated care" or "patient-centred care" or "patient centred care" or "patient-centered care" or "patient centered care" or "family nursing" or "family-centred nursing" or "family centered nursing" or "maternal child nursing" or "pediatric nursing" or "paediatric nursing" or "nursing model*" or FCC).ti,ab,kw. (26330)  41 39 or 40 (30530)  42 32 and 36 and 41 (59)  *************************** | 59 | 77 (18) |
| Ovid PsycInfo | Database: PsycINFO 1806 to present  Search Strategy:  --------------------------------------------------------------------------------  1 (((comprehensive* or integrative or systematic*) adj3 (bibliographic* or review* or literature)) or (meta-analy* or metaanaly* or "research synthesis" or ((information or data) adj3 synthesis) or (data adj2 extract*))).ti,ab,id. or ((review adj5 (rationale or evidence)).ti,ab,id. and "Literature Review".md.) or (cinahl or (cochrane adj3 trial*) or embase or medline or psyclit or pubmed or scopus or "sociological abstracts" or "web of science").ab. or ("systematic review" or "meta analysis").md. (116917)  2 premature birth/ (6267)  3 (((Preterm or premature) adj4 (Infant* or neonate* or baby or babies)) or "neonatal prematurity" or "Newborn Intensive Care Unit*" or "Neonatal Intensive Care Unit*" or NICU or "Neonatal ICU*" or "Newborn ICU*").ti,ab. (7086)  4 neonatal intensive care/ (1863)  5 2 or 3 or 4 (9845)  6 patient centered care/ (494)  7 exp family/ (331929)  8 6 and 7 (29)  9 ("family-centered" or "family-centred" or "family centered" or "family centred" or "family integrated care" or "patient-centred care" or "patient centred care" or "patient-centered care" or "patient centered care" or "family nursing" or "family-centred nursing" or "family centered nursing" or "maternal child nursing" or "pediatric nursing" or "paediatric nursing" or "nursing model*" or FCC).ti,ab. (6314)  10 8 or 9 (6325)  11 1 and 5 and 10 (15)  *************************** | 15 | 16 (1) |
| Web of Science | ((comprehensive* or integrative or systematic*) near/3 (bibliographic* or review* or literature)) or meta-analy* or metaanaly* or "research synthesis" or ((information or data) near/3 synthesis) or (data near/2 extract*) OR (review near/5 (rationale or evidence)) or "Literature Review" or cinahl or (cochrane near/3 trial*) or embase or medline or psyclit or pubmed or scopus or "sociological abstracts" or "web of science" or "systematic review" or "meta analysis" (Topic) and ((Preterm or premature) near/4 (Infant* or neonate* or baby or babies)) or "neonatal prematurity" or "Newborn Intensive Care Unit*" or "Neonatal Intensive Care Unit*" or NICU or "Neonatal ICU*" or "Newborn ICU*" (Topic) and "family-centered" or "family-centred" or "family centered" or "family centred" or "family integrated care" or "patient-centred care" or "patient centred care" or "patient-centered care" or "patient centered care" or "family nursing" or "family-centred nursing" or "family centered nursing" or "maternal child nursing" or "pediatric nursing" or "paediatric nursing" or "nursing model*" or FCC (Topic) | 114 | 151 (37) |
| CINAHL | TX ( ((comprehensive* or integrative or systematic*) n3 (bibliographic* or review* or literature)) or meta-analy* or metaanaly* or "research synthesis" or ((information or data) n3 synthesis) or (data n2 extract*) OR (review n5 (rationale or evidence)) or "Literature Review" or cinahl or (cochrane n3 trial*) or embase or medline or psyclit or pubmed or scopus or "sociological abstracts" or "web of science" or "systematic review" or "meta analysis" ) AND TX ( ((Preterm or premature) n4 (Infant* or neonate* or baby or babies)) or "neonatal prematurity" or "Newborn Intensive Care Unit*" or "Neonatal Intensive Care Unit*" or NICU or "Neonatal ICU*" or "Newborn ICU*" ) AND TX ( "family-centered" or "family-centred" or "family centered" or "family centred" or "family integrated care" or "patient-centred care" or "patient centred care" or "patient-centered care" or "patient centered care" or "family nursing" or "family-centred nursing" or "family centered nursing" or "maternal child nursing" or "pediatric nursing" or "paediatric nursing" or "nursing model*" or FCC ) | 191 | 207 (24) |
| Cochrane Database of Systematic Reviews | #1 (((Preterm or premature) near/4 (Infant* or neonate* or baby or babies)) or "neonatal prematurity" or "Newborn Intensive Care Unit*" or "Neonatal Intensive Care Unit*" or NICU or "Neonatal ICU*" or "Newborn ICU*"):ti,ab,kw 16235  #2 ("family-centered" or "family-centred" or "family centered" or "family centred" or "family integrated care" or "patient-centred care" or "patient centred care" or "patient-centered care" or "patient centered care" or "family nursing" or "family-centred nursing" or "family centered nursing" or "maternal child nursing" or "pediatric nursing" or "paediatric nursing" or "nursing model*" or FCC):ti,ab,kw 2641  #3 #1 and #2 126 | 2 | 2 (0) |
