# Supplementary material for: The global scope and components of family-centred care for preterm infants: An umbrella review
Source: PLOS Glob Public Health. 2025 Jul 3;5(7):e0004900. doi: 10.1371/journal.pgph.0004900 (PMC12225843; doi:10.1371/journal.pgph.0004900)
Supplement: S3 File — (PDF) [file pgph.0004900.s005.pdf]

PRISMA 2020 for Abstracts Checklist

Completed for: Adella J, Maraschin FG, Nagraj S. The global scope and components of family-centred care for preterm infants: an umbrella review

Section and Topic	Item #	Checklist item	Reported (Yes/No)
**TITLE**			
Title	1	Identify the report as a systematic review.	Yes
**BACKGROUND**			
Objectives	2	Provide an explicit statement of the main objective(s) or question(s) the review addresses.	Yes
**METHODS**			
Eligibility criteria	3	Specify the inclusion and exclusion criteria for the review.	Yes
Information sources	4	Specify the information sources (e.g. databases, registers) used to identify studies and the date when each was last searched.	No. This is not detailed in the Abstract due to word count limitations, but detailed in the main article, under Methods.
Risk of bias	5	Specify the methods used to assess risk of bias in the included studies.	Yes
Synthesis of results	6	Specify the methods used to present and synthesise results.	Yes
**RESULTS**			
Included studies	7	Give the total number of included studies and participants and summarise relevant characteristics of studies.	Yes
Synthesis of results	8	Present results for main outcomes, preferably indicating the number of included studies and participants for each. If meta-analysis was done, report the summary estimate and confidence/credible interval. If comparing groups, indicate the direction of the effect (i.e. which group is favoured).	Yes
**DISCUSSION**			
Limitations of evidence	9	Provide a brief summary of the limitations of the evidence included in the review (e.g. study risk of bias, inconsistency and imprecision).	No. This is not detailed in the Abstract due to word count limitations, but detailed in the main article, under Discussion.
Interpretation	10	Provide a general interpretation of the results and important implications.	Yes
**OTHER**			
Funding	11	Specify the primary source of funding for the review.	No. This is not detailed in the Abstract due to word

PRISMA 2020 for Abstracts Checklist

Completed for: Adella J, Maraschin FG, Nagraj S. The global scope and components of family-centred care for preterm infants: an umbrella review

Section and Topic	Item #	Checklist item	Reported (Yes/No)
			count limitations, but detailed in the main article, under Funding.
Registration	12	Provide the register name and registration number.	No. This manuscript is not registered.

From: Page MJ, McKenzie JE, Bossuyt PM, Boutron I, Hoffmann TC, Mulrow CD, et al. The PRISMA 2020 statement: an updated guideline for reporting systematic reviews. BMJ 2021;372:n71. doi: 10.1136/bmj.n71. This work is licensed under CC BY 4.0. To view a copy of this license, visit https://creativecommons.org/licenses/by/4.0/
